# Enhanced Cytotoxic Activity of Mitochondrial Mechanical Effectors in Human Lung Carcinoma H520 Cells: Pharmaceutical Implications for Cancer Therapy

**DOI:** 10.3389/fonc.2018.00514

**Published:** 2018-11-13

**Authors:** Sergio González Rubio, Nuria Montero Pastor, Carolina García, Víctor G. Almendro-Vedia, Irene Ferrer, Paolo Natale, Luis Paz-Ares, M. Pilar Lillo, Iván López-Montero

**Affiliations:** ^1^Departamento de Química Física, Universidad Complutense de Madrid, Madrid, Spain; ^2^Instituto de Investigación Hospital 12 de Octubre (i+12), Madrid, Spain; ^3^Departamento de Química Física Biológica, Instituto de Química-Física “Rocasolano” (CSIC), Madrid, Spain; ^4^Centro Nacional de Investigaciones Oncológicas (CNIO), Madrid, Spain; ^5^Departamento de Medicina, Universidad Complutense de Madrid, Madrid, Spain; ^6^Ciberonc, Madrid, Spain

**Keywords:** NAO, mitochondrial targeting, membrane adhesion, cancer therapy, NSCLC cells, FLIM, phasor analysis

## Abstract

Cancer cell mitochondria represent an attractive target for oncological treatment as they have unique hallmarks that differ from their healthy counterparts, as the presence of a stronger membrane potential that can be exploited to specifically accumulate cytotoxic cationic molecules. Here, we explore the selective cytotoxic effect of 10-*N*-nonyl acridine orange (NAO) on human lung carcinoma H520 cells and compare them with healthy human lung primary fibroblasts. NAO is a lipophilic and positively charged molecule that promotes mitochondrial membrane adhesion that eventually leads to apoptosis when incubated at high micromolar concentration. We found an enhanced cytotoxicity of NAO in H520 cancer cells. By means Fluorescence lifetime imaging microscopy (FLIM) we also confirmed the formation of H-dimeric aggregates originating from opposing adjacent membranes that interfere with the mitochondrial membrane structure. Based on our results, we suggest the mitochondrial membrane as a potential target in cancer therapy to mechanically control the cell proliferation of cancer cells.

## Introduction

Cancer remains one of the most common causes of death worldwide. In particular, lung cancer is usually detected at later stages of development (1). Additionally, the appearance of drug resistance during the course of treatment often leads to unsatisfying outcomes and a poor prognosis of patients (2) limited to a 5 years survival rate (3).

Traditional chemotherapeutics interfere with replication or cell division to prevent cell growth, increase cell death and restrict the spreading of the cancer (4). Side effects of traditional cancer treatments as surgery, chemotherapy or radiation therapy are significant as many healthy cells are killed during the treatment, due to the lack of target specificity. With the aim to specifically target only cancer cells, a new generation of cancer treatment has been developed recently known as the targeted cancer therapy (5), where pharmacological agents (monoclonal antibodies, small molecule inhibitors or immunotoxins) that interfere with specific signaling proteins involved in tumor genesis are used (5). For instance, the epidermal growth factor receptor (EGFR) represents currently a major target in lung cancer therapy. However, the development of acquired resistance is now well recognized for promising monoclonal antibodies acting as EGFR inhibitors (6, 7). A new promising strategy for cancer therapy focuses on mitochondria as they participate as key regulators in the apoptosis. Mitochondria of cancer cells exhibit unique and multiple characteristics that differ from their healthy counterparts. Among them, a stronger (more negative) mitochondrial membrane potential (8) that suggests an underlying increased accumulation of cytotoxic cationic molecules within cancer cells (9–13). The compounds directed to mitochondria and able to interfere with the mitochondrial function, become promising antitumor agents (4, 14–16) and may represent a more selective and effective option for therapy.

As a proof-of-concept, we explore here the selective cytotoxic effect of the compound 10-*N*-nonyl acridine orange (NAO) on human lung carcinoma H520 cells. NAO is a lipophilic fluorescent molecule used as a mitochondrial marker that stains the inner mitochondrial membrane (IMM) (11). NAO was previously shown to be cytotoxic when present at low milimolar concentrations (17) and we have recently shown the underlying molecular mechanisms of this cytotoxicity. The formation H-aggregates of NAO molecules originating from opposing adjacent membranes elicits interbilayer adhesion of mitochondria and interferes with mitochondrial dynamics leading to apoptosis (18).

We demonstrate the specific cytotoxic activity of NAO in H520 human lung carcinoma cells exposed at NAO concentration that are not harmful for healthy human lung primary fibroblast (HLPF). H520 cells represent a paradigmatic non-small cell lung cancer (NSCLC) cells and they have proven to be a powerful tool for cancer research. We also identify by means of Fluorescence life time imaging microscopy (FLIM) the presence of long-lifetime H-dimers of NAO, generally formed when present at high local concentration.

## Results

### NAO exhibits a strong cytotoxic activity in H520 cancer cells

**Figure 1:**
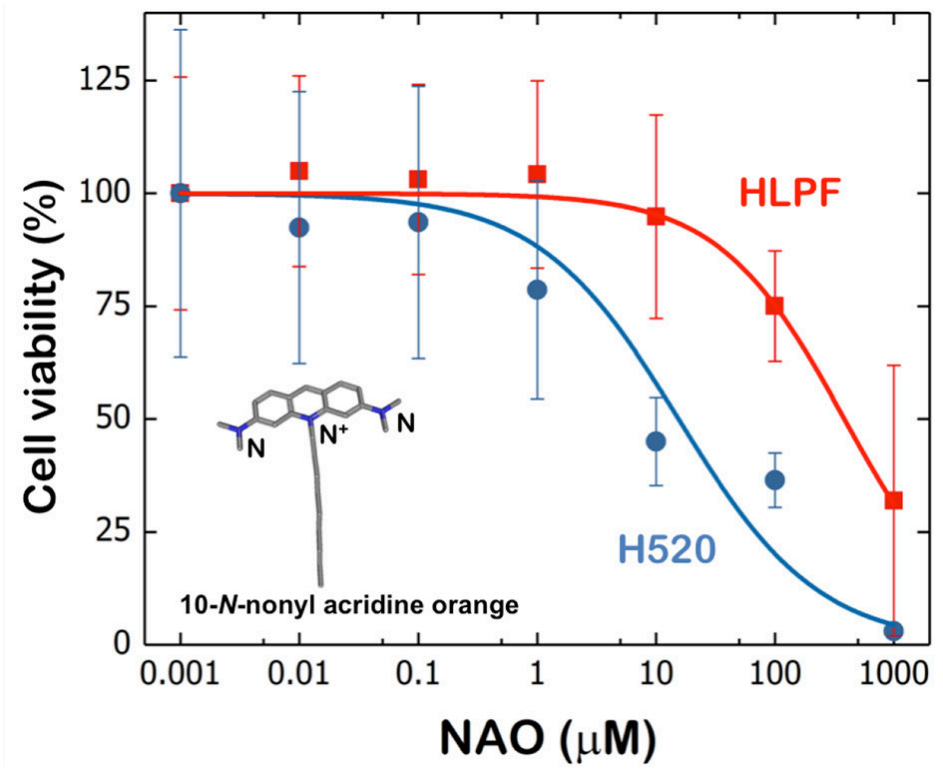
Cell viability of HLPF and H520 cells upon NAO incubation. The experimental data were satisfactorily fitted by a classical sigmoidal curve that is obtained with the Hill model (19). The Hill model is based on the equation $C_{Hill}=\;C_{0}+\frac{C_{\infty }-C_{0}}{1+{\left(\frac{CC_{50}}{C}\right)}^{H}}$, which describes the cytotoxic effect C_Hill_ obtained at a given concentration C; where CC_50_ is the relative 50% cytotoxic concentration reducing cell viability by 50%, H is the Hill exponent, C_∞_ is the maximum cytotoxicity and C_0_ is the viability in the absence of NAO. **Inset:** Chemical structure of 10-*N*-nonyl acridine orange (NAO).

To determine the enhanced cytotoxicity of NAO in cancer cells, H520 cancer cells and healthy HLPF (control) were exposed for 60 min to increasing amounts of NAO (1 nM−1 mM) and cell viability was determined by the MTT assay (Figure 1). The viability of H520 starts to be compromised in the presence of 0.1 μM and cells were severely killed in the presence of NAO at concentrations up to 1 mM. The cell viability of HLPF is not affected until the presence of 10 μM of NAO and shows a survival of approximately 30% in the presence of 1 mM of NAO. The calculated CC_50_ values vary 25 fold with ≈15 μM for H520 and ≈385 μM for HLPF (Figure 1). Alternative cationic dyes 1,1′-Dioctadecyl-3,3,3′,3′-Tetramethylindotricarbocyanine Iodide (DiR) and Tetramethyl rhodamine methyl ester (TMRM) were also tested for cell toxicity. DiR is long-chain lipophilic carbocyanine dye (20) and TMRM is a dye used for monitoring the mitocondrial membrane potential (21). The obtained CC_50_ values of these dyes vary from ≈3 × 10^2^ μM for H520 cells to > mM for HLPF cells (Figure S1). The toxicity of DiR and TMRM were only observed in cancer cells and at one order of magnitude higher than observed for NAO. Hereinafter we focus on NAO because of its enhanced cytotoxicity.

**Figure 2:**
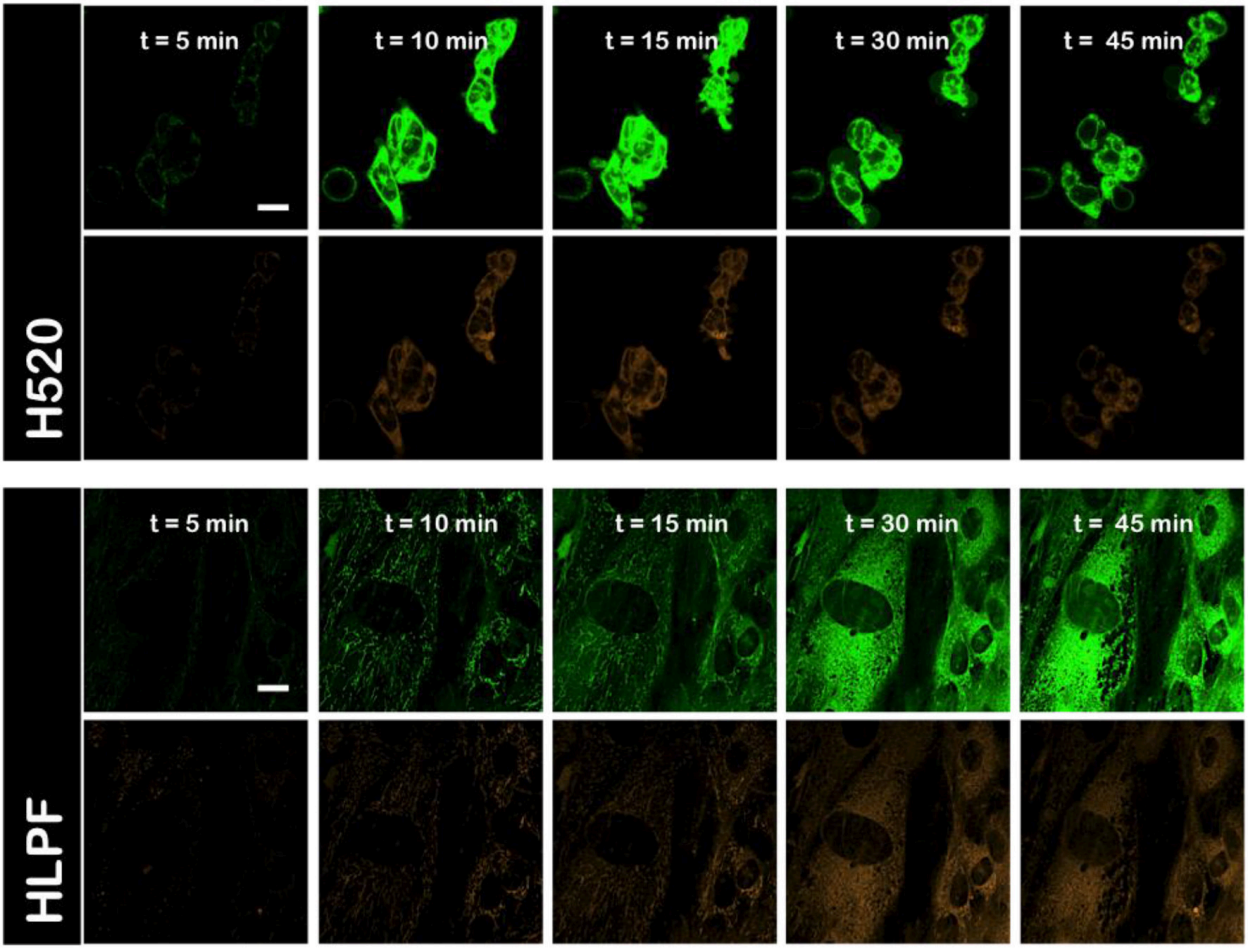
Interbilayer NAO dimers elicit cytotoxicity in HLPF and H520 cells. Confocal fluorescence micrographs (green channel, λ_exc_ = 488 nm and red channel, λ_exc_ = 561nm) of HLPF and H520 mitochondria in the presence of 5 μM of NAO. NAO induces apoptosis and the spectral shift from green to red, indicative for the formation of interbilayer NAO dimers (See main text for details). Scale bars are 10 μm.

Next, we measured the conformational arrangement of NAO in H520 and HLPF cells by means of fluorescence confocal microscopy. The incubation with high concentration of NAO leads to cell death accompanied with a green-to-red emission shift where the maximum emission wavelength at λ_em_ = 525 nm shifts to 640 nm due to the formation of supramolecular aggregates (22) that result from the π-π interactions between the stacked acridine orange moieties (23, 24). In agreement with the previous literature and our own observations (11, 18) the incubation of NAO at low nM concentrations merely stains the mitochondrial network of both H520 and HLPF cells, but does not exhibit visual signs of cytotoxicity (Figure S2). The low red shift of NAO observed in HPLF cells did not induced apoptosis, even for long incubation times (up to 48 h). In contrast, at high μM concentrations, the mitochondria of H520 immediately stained and cells entered apoptosis that can be appreciated by the global morphological changes as membrane blebbing or cell shrinkage (Figure 2). As previously shown, this morphological remodeling of the mitochondrial network into spherical liposomal appearance is simultaneously accompanied by the red shift of NAO and a direct consequence of the formation of supramolecular NAO zippers among adjacent mitochondrial membranes (Figure 2) (18). At high concentrations, also HLPF control cells were compromised and both the red shift and the morphological changes of mitochondria were appreciated at longer incubation times (Figure 2).

### The cytotoxic effect of NAO in H520 and HLPF cells originates from the supramolecular assembly of NAO dimers in mitochondrial membranes

Time-resolved fluorescence imaging allowed us to characterize the molecular basis of the NAO induced cytotoxicity. Within eukaryotic cells and depending on the administered concentration of NAO, three spectroscopic molecular species of NAO can be observed in the mitochondrial membranes. The molecular species are characterized by different excited-state lifetimes of 0.2 ns (very short excited-state species), 2.0 ns (intermediate excited-state species), and 10 ns (long excited-state species). The very short species corresponds to the self-quenched clustered form of NAO, the intermediate species to the monomeric form and the long excited-state species to the red-shifted dimeric form (18). Note that, the 10-ns lifetime NAO species was only detected in the red channel #1; whereas the 2.0-ns and 0.2-ns NAO species were detected in both emission channels, but mainly in the green channel #2.

**Figure 3:**
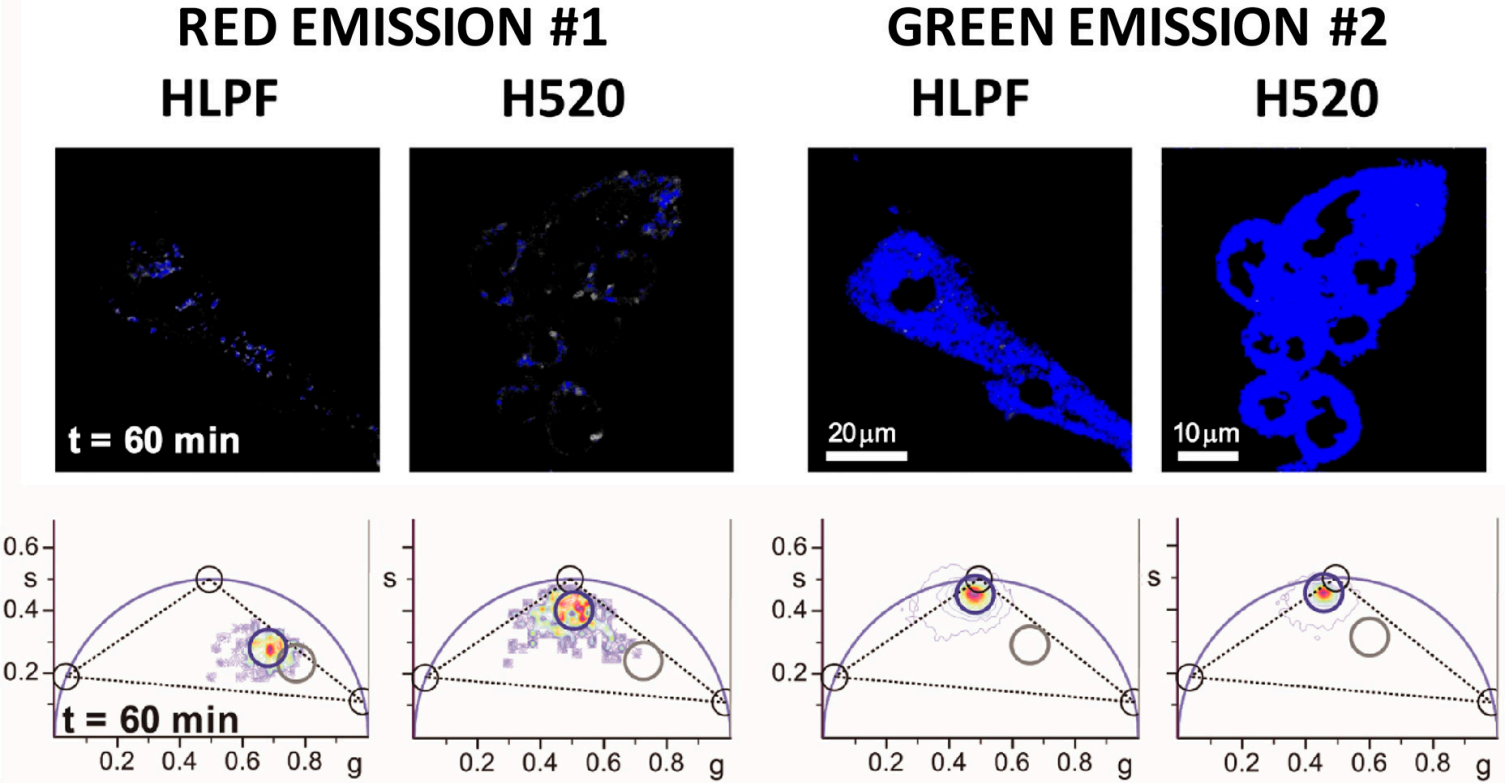
Spatial distribution of NAO molecular species in HLPF and H520 cells. Basal *XY* sections of representative groups of cells treated with 10 nM NAO for 60 min and their corresponding phasor plots showing regions enriched in intermediate-lifetime NAO monomers (blue cursor; as detected in channel #1 and channel #2). Gray cursors show cell autofluorescence. Black cursors on the phasor plot represent 100% pure NAO species: NAO antiparallel H-dimers (τ = 10 ns), monomeric NAO (τ = 2.0 ns), and self-quenched NAO aggregates (τ = 0.2 ns). Black dashed lines joining the three pure species phasors form a triangle that would contain phasors from all possible intensity-weighted combinations of the three NAO molecular species. Pixels corresponding to the cluster of phasors enclosed by the colored circles are highlighted on the intensity image using the same color. λ_exc_ = 850 nm, red channel (#1) FF01 685/40, green channel (#2) FF02 520/35, dichroic filter FF560-Di01 (Semrock, Germany), 1.2 ms/pixel.

**Figure 4:**
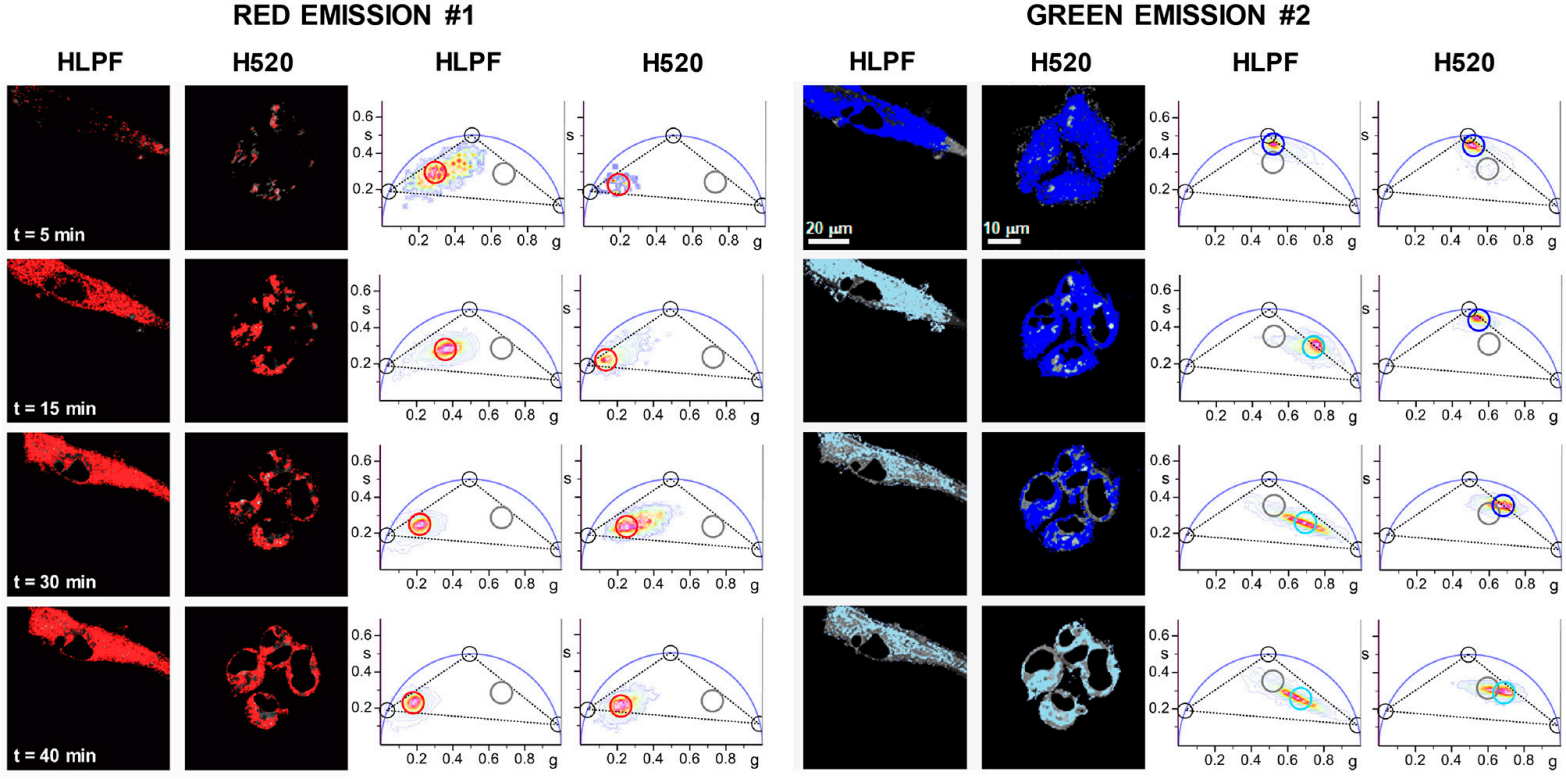
Spatial distribution of H-dimers in HLPF and H520 cells. Basal *XY* sections of representative groups of cells treated with 5 μM NAO at different times. Their corresponding phasor plots show regions enriched in long-lifetime NAO antiparallel H-dimers (red cursor; as detected in channel #1), intermediate-lifetime NAO monomers (blue cursor; as detected in channel #2) and the short-lifetime NAO self-quenched clusters (light blue cursor; as detected in channel #2). Gray cursors show cell autofluorescence. Black cursors on the phasor plot represent again 100% pure NAO species: NAO antiparallel H-dimers (τ = 10 ns), monomeric NAO (τ = 2.0 ns), and self-quenched NAO aggregates (τ = 0.2 ns). Black dashed lines joining the three pure species phasors form a triangle that would contain phasors from all possible intensity-weighted combinations of the three NAO molecular species. Pixels corresponding to the cluster of phasors enclosed by the colored circles are highlighted on the intensity image using the same color. λ_exc_ = 850 nm, red channel (#1) FF01 685/40, green channel (#2) FF02 520/35, dichroic filter FF560-Di01 (Semrock, Germany), 1.2 ms/pixel.

Two-photon FLIM-phasor images of a representative group of H520 and HLPF cells incubated for 60 min with 10 nM NAO show the presence and the quantification of the three species. All three lifetimes (10, 2.0, and 0.2 ns) lie on the universal phasor circle (black cursors) (25). In the low nM concentration regime (Figure 3), the phasor clusters determined for the channel #1 and #2 FLIM images correspond to nearly pure NAO monomer molecules for both cell lines. Note that the lower accumulation of NAO in HLPF cells leads the phasor cluster of the channel #1 to overlap the phasor cluster corresponding to autofluorescence. When both H520 and HLPF cells are incubated for short times (up to 60 min) with NAO at high μM concentrations the phasor cluster correspond to a mixture of the three species as indicated by the localization of the phasor cluster inside the three species triangle in channel #1 and #2 (Figure 4).

Though the population of NAO (2.0 ns) monomers species predominates (blue cursor and blue color in the phasor color map) in the early stages of the interaction, the (10 ns) long excited-state NAO species becomes predominant (in red channel #1; red cursor and red color in the phasor color map) and the (0.2 ns) self-quenched population species emerges (blue channel #2; light blue cursor and light blue color in the phasor color map) during the progress of NAO accumulation. As previously shown (18) the formation of red-shifted NAO-dimers indicates the supramolecular assembly of molecular zippers between stacked NAO from opposing bilayers producing membrane remodeling in mitochondria that compromise the survival of the cell (Figure 2). This molecular mechanism by which the widely used mitochondrial dye NAO exhibits cytotoxicity is enhanced in NSCLC H520 cells as compared to HLPF cells (Figure 1).

## Discussion

The primary function of mitochondria is to provide the chemical energy to eukaryotic cells in the form of adenosine triphosphate (ATP). As this function is essential for cell viability, mitochondria are being explored as a promising target for therapeutic purposes. The fact that mitochondria of cancer cells exhibit characteristics different from healthy cells, allow the design of new strategies focusing on enhanced selectivity and reduced acquired drug resistance. Beyond its well-established role in cellular energetics, mitochondria are key controllers of apoptosis (26). Pro-apoptotic factors, such as cytochrome c, reside in the mitochondrial intermembrane space and are liberated to the cytoplasm during apoptosis. This activates caspase proteases and the subsequent cleavage of structural and regulatory proteins in the cytoplasm and the nucleus (27). New cancer therapies aim to specifically induce apoptosis through pharmacological agents acting on mitochondria and promoting mitochondrial failure or damage (14). Cancer cells exhibit a stronger (more negative) mitochondrial membrane potential, a particular feature that opens the way to control of cell proliferation and survival by means of regulation of the mechanical properties of mitochondrial membranes. This strong membrane potential of mitochondria allows the accumulation of cationic lipophilic molecules within the mitochondrial matrix at non-effective concentrations of normal cells. This rational leads to the design and synthesis of mitochondrial dyes (18, 28), such as NAO, a lipophilic and positively charged fluorescent dye able to diffuse spontaneously into membrane environments (17). The incubation with concentrations in the μM range of NAO induces cytotoxicity (26). In contrast to other cationic dyes that accumulate in mitochondria (DiR and TMRM), it is only required a lower concentration of NAO to produce fatal consequences for cell viability (17). The ability of NAO to form supramolecular stacks is provided by the acridine orange moiety (23), but the presence of the aliphatic chain enhances the partitioning of NAO into the membrane and promotes its cytotoxic mechanism (18). NAO dimers from opposite membranes trigger their adhesion and cause severe mechanical alterations of mitochondrial dynamics, promoting mitochondrial membrane remodeling. As a result, mitochondria lose their characteristic ultrastructure and form double-membrane vesicles comprising the outer and inner membranes (18, 29). The stronger cytotoxicity of NAO on carcinoma H520 cells is compatible with a higher accumulation of NAO into cancer mitochondria producing cell death at lower effective dose compared to non-cancerous cells (Figure 1). We also show a similar accumulating effect in cancer cells for the other cationic dyes tested (Figure S1).

**Figure 5:**
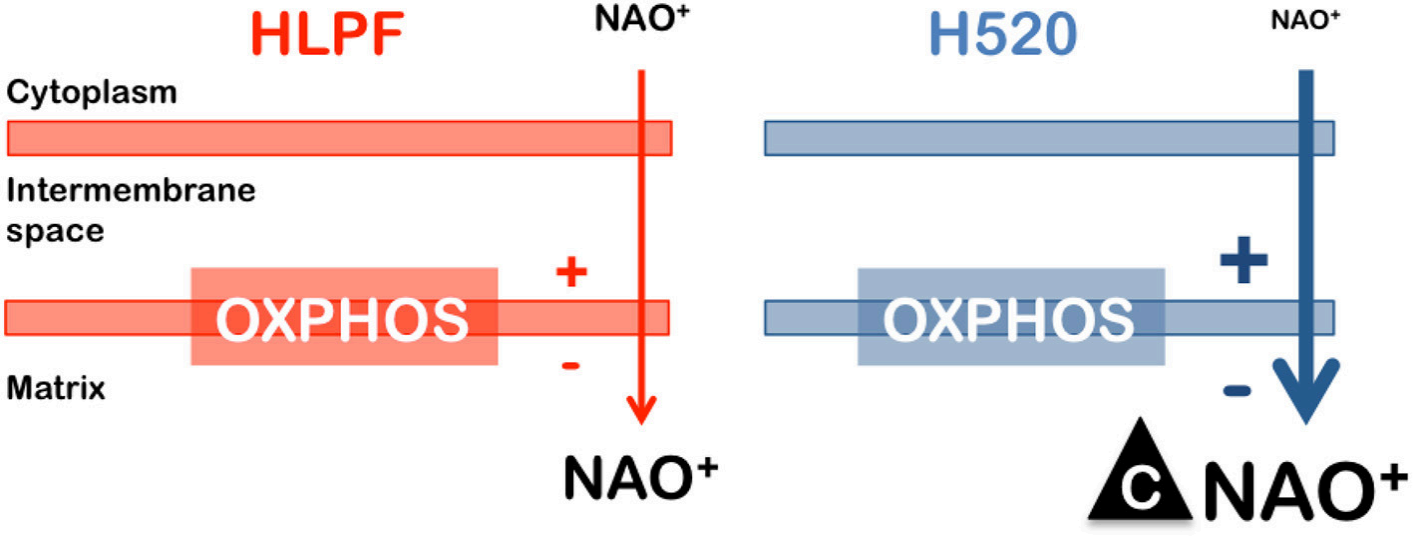
Strategy focusing on enhanced selectivity for cancer cells. The negative membrane potential of healthy mitochondria (HLPF cells) enables lipophilic and positively charged molecules (NAO) to accumulate in the inner membrane. A more negative membrane potential in cancer cells (H520 cells) triggers an enhanced accumulation of NAO leading to cytotoxicity.

Note that the mitochondrial membrane potential established by the Nernst equation:

$$\begin{array}{l} E=\;2.3\frac{RT}{zF}\;log\left[\frac{{\left[NAO\right]}_{o}}{{\left[NAO\right]}_{i}}\right] \end{array}$$

where *E* is the membrane potential, [*NAO*]_*o*_ is the cytosolic concentration of NAO, [*NAO*]_*i*_ is the mitochondrial concentration of NAO, *R* is the ideal gas constant, *T* is the temperature, *z* is the charge of the dye and *F* is the Faraday's constant. Thus, a stronger (e.g., two-fold) mitochondrial potential in cancer cells (21, 29) will result in a ten-fold accumulation of the cationic probe within the cancer cell (Figure 5). Additional factors as an increased mitochondrial mass (30) or a different composition of negatively charged lipids as cardiolipin (31) can also control the enhanced selectivity of lipophilic and positively charged molecules. To reliably ground the physico-chemical mechanisms that drive the enhanced NAO accumulation in cancer mitochondria the direct measurement of both the membrane potential and the absolute concentration of NAO in living cells is required (32).

Anticancer drugs that directly target mitochondria might have the potential to bypass the drug-resistance acquired by cancer cells during chemotherapy treatment. In particular, novel targeted therapies must be based on physical mechanisms at the mesoscale, the adhesion of mitochondrial membranes in our case, instead of interfering with the molecular biochemical signals that can be easily reprogrammed by the cell. The mechanical targeting proposed here may provide a unique tool to circumvent the acquired survival mechanisms and may be effective in otherwise resistant forms of cancer. The proof-of-concept still needs to explore the therapeutic window with more NSCLC cell lines including those that acquired resistance during chemotherapy and more important with other kind of healthy cells, in particular for those more susceptible to mitochondrial functioning. Also, *in vivo* experiments with murine models are essential for a complete validation of the approach.

## Materials and methods

### Chemicals

10-*N*-nonyl acridine orange (NAO), 1,1′-Dioctadecyl-3,3,3′,3′-Tetramethylindotricarbocyanine Iodide (DiR) and Tetramethyl rhodamine methyl ester (TMRM) were supplied by Thermofisher. Ultrapure water was produced from a Milli-Q unit (Millipore, conductivity below 18 MΩ cm).

### Cell culture

The cell line of human lung primary fibroblast (HLPF) was provided by Pr. Alejandro Sweet-Cordero from University of California San Francisco (UCSF) under the terms of a Material Transfer Agreement; and no ethical approvals were required for their use in this study as per local legislation and national guidelines. HLPF cells were maintained in DMEM + GlutaMax-I, supplemented with 10% fetal bovine serum (FBS), penicillin (50 U/mL) and streptomycin (50 μg/mL) (Thermofisher) and human lung tumor cells (H520) were maintained in RPMI-1640 medium, supplemented with 10% fetal bovine serum (FBS), penicillin (50 U/mL) and streptomycin (50 μg/mL) (Thermofisher). All cells were maintained in a humidified incubator at 37°C and 5% CO_2_ atmosphere (Forma Steri-Cycle Themofisher; 5% CO_2_). Cells were plated in 75 cm^2^ flasks (Thermofisher) and were passaged when reaching 95% confluence, by gentle trypsinization 0.05% trypsin/0.53 mM EDTA; Invitrogen Life Technologies).

### Cell viability

Cell viability was evaluated by using the methylthiazoletetrazolium assay (MTT; Sigma-Aldrich). All experimental conditions were performed in quadruplicate. Cells were seeded in 96-well flat-bottom plates at 5,000 cells/well. After 24 h, the medium was replaced with 100 μl medium with different concentrations of NAO, DiR and TMRM (1 nM−1 mM). After 60, 90, 120, and 150 min, each well was replaced with 100 μl MTT solution (5 mg/ml). After 2 h incubation at 37°C, 5% CO_2_, each well was replaced with 100 μl DMSO (Sigma-Aldrich). The cell viability was determined by measuring the absorbance at 570 nm using a spectrophotometer (Multiskan^TM^ FC, Thermofisher). Results are presented as the percentage survival in relation to untreated control cells.

### Confocal microcopy

Confocal microscopy images of cells were collected at 37°C with a Nikon Ti-E inverted microscope equipped with a Nikon C2 confocal scanning confocal module, 488 and 561 nm continuous lasers, emission bandpass filters, and a Nikon Plan Apo 100 × NA 1.45 oil immersion objective. Both H520 and HLPF cells were seeded at 3 × 10^4^ cells per cm^2^ in a four-chamber Lab-Tek® slide (Thermofisher) and incubated with complete DMEM for HLPF cells and RPMI for H520 cells, both for 24 h at 37°C. Prior to confocal fluorescence imaging, cells were supplemented with NAO (5 nM or 5 μM).

### Two-photon fluorescence lifetime imaging

Two-photon, two-color (red channel #1 and green channel #2) fluorescence-lifetime imaging (2P-FLIM) of XY sections of cells was carried out on a MicroTime200 system (PicoQuant, Germany) equipped with a mode-locked, femtosecond-pulsed Ti:Sapphire laser (Mai-Tai, Spectra Physics, CA) operating at a repetition rate of 80 MHz, horizontally polarized, and tuned to 850 nm; an Olympus IX71 inverted microscope mounted with a 60X water-immersion objective NA1.2; a piezo XY-scanning table and two single-photon counting avalanche diodes (τ-SPAD, PicoQuant, Germany), and a PicoHarp 300 PC-board (PicoQuant, Germany), synchronized with the excitation laser pulses using the Time-Tagged Time-Resolved (TTTR) detection mode at 23°C. The TTTR mode allows the recording of every individual fluorescence photon from each pixel, together with its timing and emission color (channel #1, channel #2). The acquisition time per pixel accounted for 1.2 ms, resulting in an image overall acquisition time of 180 s. Once the acquisition of the image was finished, all the detected photons per pixel were used to build steady-state fluorescence intensity images or to produce Fast FLIM (Figure S3) and FLIM phasor images using the ps-temporal resolution of the system, SymphoTime 64 software (Fast FLIM; PicoQuant, Germany), and SimFCS software (Phasor Analysis) developed at the Laboratory of Fluorescence Dynamics (LFD, UC Irvine). The filters used in this study were all from Semrock (Germany). Red channel (#1): FF01-685/40; green channel (#2): FF01-520/35; with a dichroic beam splitter FF560-Di01. Although in the red channel #1 we mostly select red NAO aggregates, a non-negligible bleed through from NAO monomers may exist, particularly when the monomer NAO population is the majority. The excitation power was lower than 1 mW at the sample, and it was adjusted using a variable optical attenuator LS-107AT (Lasing, S.A. Spain) to achieve counting rates below 10^6^ photons/s. Both H520 and HLPF cells were seeded at 2 × 10^4^ cells per cm^2^ in an 8-chamber Lab-Tek® slide (Thermofisher) and incubated with complete RPMI 1640 or DMEM for 24/36 h at 37°C. Prior to FLIM imaging at the micro-spectrometer, cells were washed with Tyrode-glucose buffer (NaCl 145 mM, KCl 4 mM, MgCl_2_ 1 mM, CaCl_2_ 1.8 mM, HEPES-Na 10 mM, glucose 10 mM, pH 7.4) and supplemented with NAO (10 nM or 5 μM).

## Author contributions

IL-M designed research. SGR, NM, CG, VA-V, and MPL performed research. IF and LP-A contributed new reagents. SGR, NM, CG, MPL, and IL-M analyzed data. PN, LP-A, MPL, and IL-M wrote the paper.

### Conflict of interest statement

The authors declare that the research was conducted in the absence of any commercial or financial relationships that could be construed as a potential conflict of interest.
