# Supplementary material for: Enhanced Cytotoxic Activity of Mitochondrial Mechanical Effectors in Human Lung Carcinoma H520 Cells: Pharmaceutical Implications for Cancer Therapy
Source: Front Oncol. 2018 Nov 13;8:514. doi: 10.3389/fonc.2018.00514 (PMC6242888; doi:10.3389/fonc.2018.00514)
Supplement: Supplementary file 1 [file Data_Sheet_1.docx]

**Supplementary Material**

**
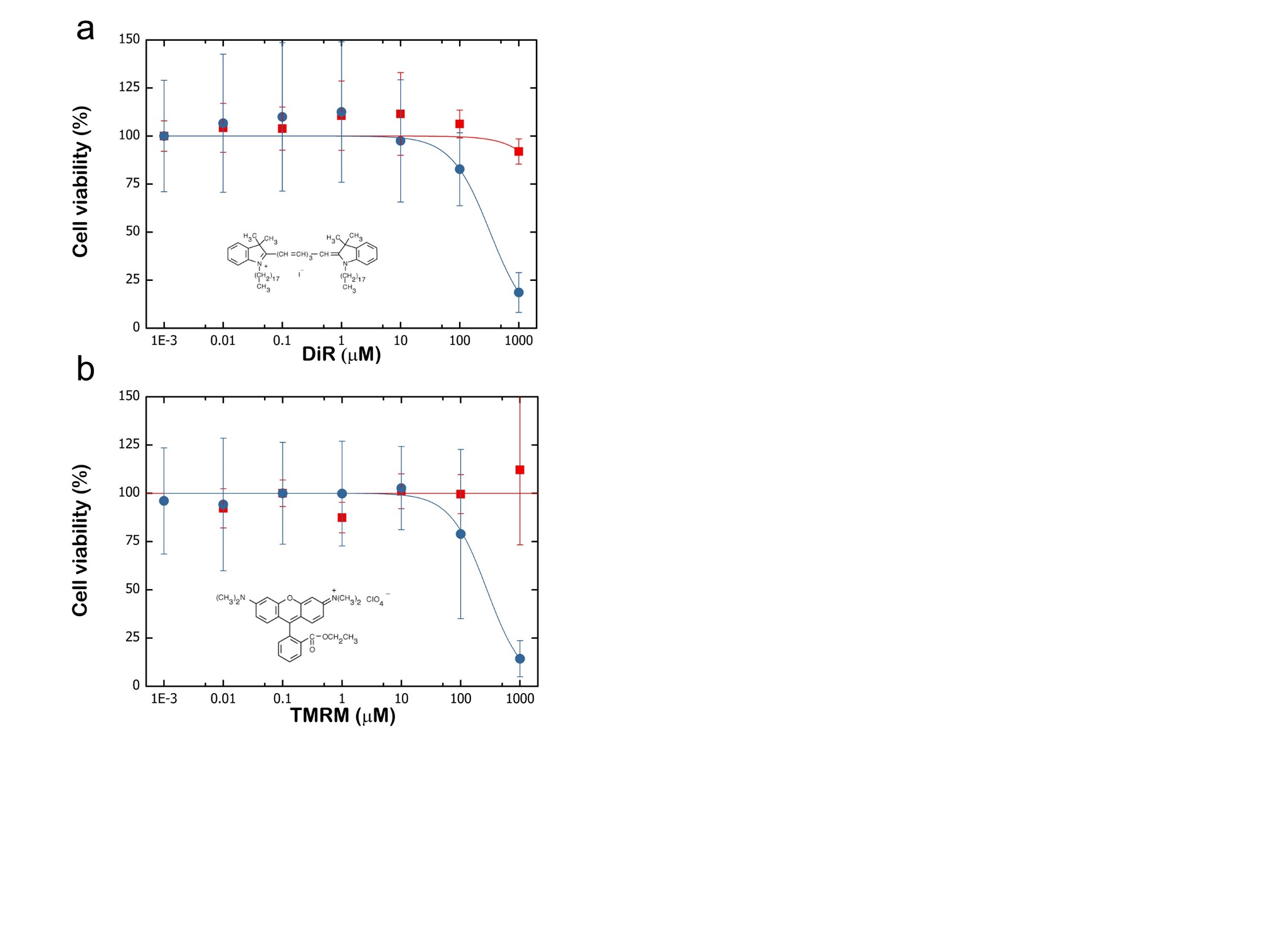
**

**Figure S1. Cell viability of HLPF and H520 cells upon a) DiR and b) TMRM incubation.** The experimental data were fitted to the Hill equation$C_{Hill}= C_{0}+\frac{C_{\infty}-C_{0}}{1+\left( {{CC}_{50}}/C \right)^{H}}$ (see main text). The obtained ${CC}_{50}$values in H520 cells were ${CC}_{50}^{DiR}$ = 327 μM and ${CC}_{50}^{TMRM}$ = 277 μM for DiR and TMRM respectively. **Inset a and b:** Chemical structure of 1,1'-Dioctadecyl-3,3,3',3'-Tetramethylindotricarbocyanine Iodide (DiR) and Tetramethyl rhodamine methyl ester (TMRM) respectively.

**
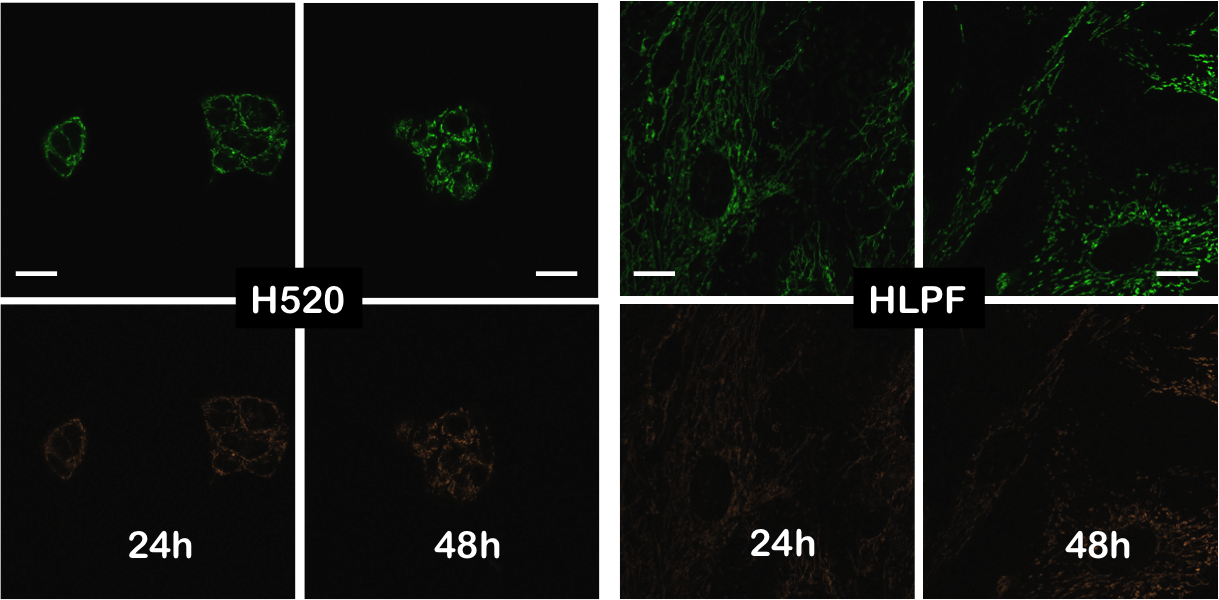
**

**Figure S2. HLPF and H520 mitochondria staining by NAO.** Confocal fluorescence micrographs (green channel, λ_exc_ = 488nm and red channel, λ_exc_ = 561nm) of HLPF and H520 mitochondria in the presence of 5 nM of NAO. At low nM concentrations, NAO stains the mitochondrial network and does not elicit cell death. Scale bars are 10 μm.

**
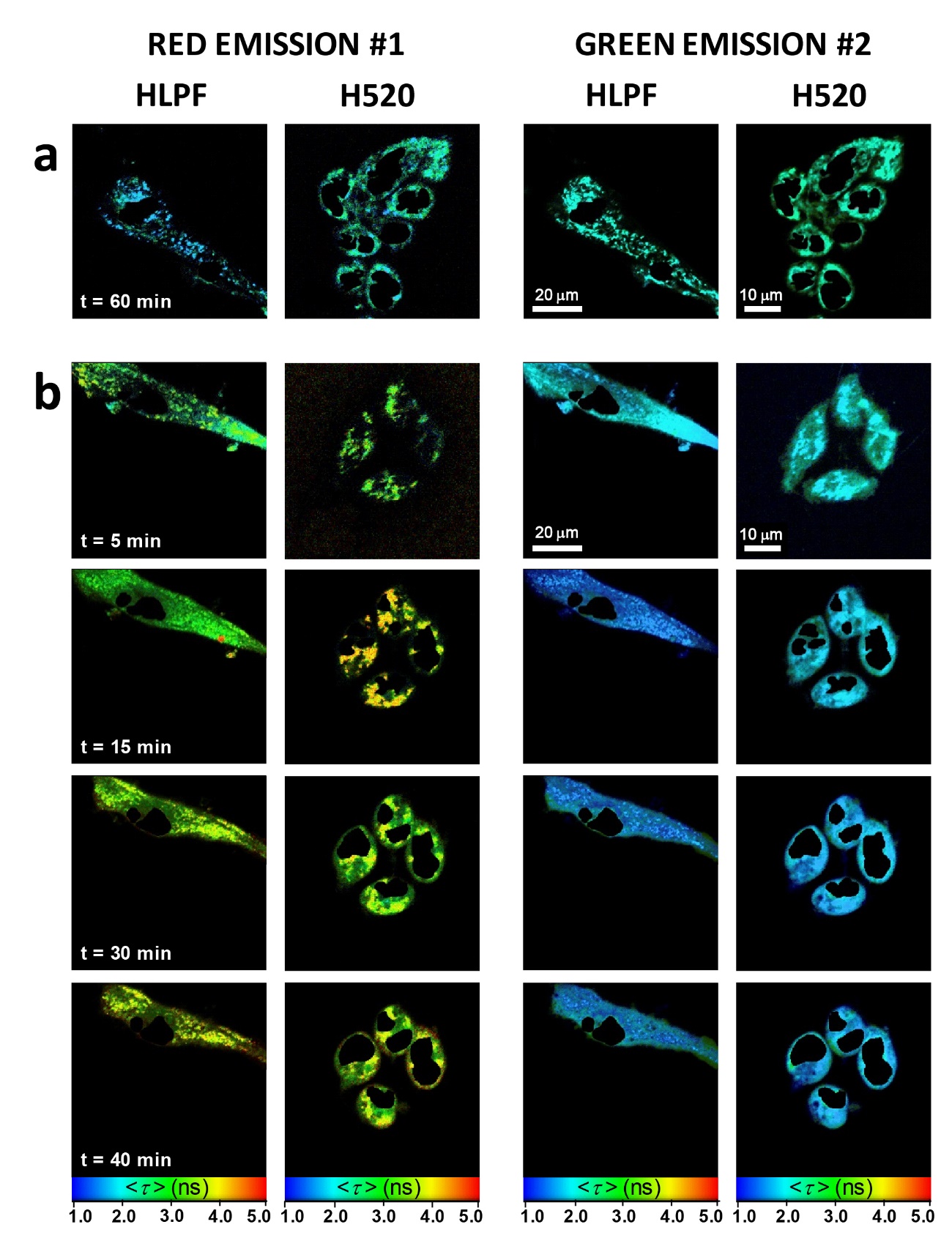
**

**Figure S3.** **Fast FLIM images** **of NAO in HLPF and H520 cells.** Basal *XY* sections of representative groups of cells treated with **(a)** 10 nM NAO for 60 min and **(b)** 5 μM NAO at different times. First and second columns: Intensity-weighted Fast FLIM images of HLPF and H520 cells acquired in the red channel (#1). Third and fourth columns: Intensity-weighted Fast FLIM images of HLPF and H520 cells acquired in the green channel (#2). Fast FLIM rainbow color scale (1–5 ns). *λ*_exc_ = 850 nm, red channel (#1) FF01 685/40, green channel (#2) FF02 520/35, dichroic filter FF560-Di01 (Semrock, Germany), 1.2 ms/pixel. Fast FLIM images show a nearly uniform distribution of intermediate average lifetime species in both channels at low NAO concentration (a) and at short times at high NAO concentration (b). Upon incubation with high micromolar concentration of NAO (b), Fast FLIM (#1) images show a progressive appearance of longer average excited-state lifetimes (<* *>), whereas Fast FLIM (#2) images show a progressive appearance of shorter average excited-state lifetimes (<* *>). Fast FLIM images obtained with the SymphoTime software (PicoQuant, Germany) use the average photon arrival time per pixel as an indicator of the intensity averaged excited-state lifetime. Note that when the experimental time window is of the same order of magnitude than the fluorophore lifetimes, the average lifetimes determined from Fast FLIM are found to be always lower than the true value. Here, we used Fast FLIM images only in a qualitative way to discriminate regions of interest enriched in the different NAO molecular species (see Methods). A detailed global analysis of the picosecond time-resolved decays from different regions of interest (ROIs) by a multiexponential function revealed the existence of three NAO molecular species with excited-state lifetimes of 10, 2.0, and 0.2 ns in varying relative proportions depending on the selected ROI and emission channel (data not shown).
